# Neurological symptoms in a patient with isolated adrenocorticotropin deficiency: case report and literature review

**DOI:** 10.1186/s12902-015-0082-6

**Published:** 2016-01-12

**Authors:** Yukihiro Goto, Kazunori Tatsuzawa, Kazuyasu Aita, Yuichi Furuno, Takuya Kawabe, Kei Ohwada, Hiroyasu Sasajima, Katsuyoshi Mineura

**Affiliations:** Department of Neurosurgery, Kyoto Prefectural University Graduate School of Medicine, Kawaramachi-Hirokoji, Kamigyo-ku, Kyoto 602-8566 Japan

**Keywords:** Isolated ACTH deficiency, Neurological symptom, Gait disturbance, Idiopathic normal pressure hydrocephalus

## Abstract

**Background:**

Isolated adrenocorticotropic hormone (ACTH) deficiency is a pituitary disorder characterized by reduction only in the secretion of ACTH. Although the underlying mechanism remains to be elucidated, numbers of cases with this entity have been increasing. We experienced a case presenting with gait disturbance necessitating differential diagnosis from idiopathic normal pressure hydrocephalus (iNPH).

**Case presentation:**

A 69-year-old female with a complaint of difficulty walking and suspected to have iNPH at a prior hospital was referred to our department. For the prior three years, she had suffered from a progressive gait disturbance. Magnetic resonance imaging (MRI) revealed global ventricular dilatation. The typical features of the gait in iNPH cases were all identifiable. Neuropsychological dementia scale tests showed deterioration. However, the major feature of a disproportionately enlarged subarachnoid-space on MRI was not obvious. The patient developed progressively worsening fatigue during hospitalization. Her symptoms resembled those of hypothalamic-pituitary tumor patients. Serum ACTH and cortisol levels were low. While corticotrophin releasing hormone stress tests showed no response, other stress tests using thyrotropin releasing hormone, luteinizing hormone releasing hormone, and growth hormone releasing hormone yielded normal responses, indicating a diagnosis of isolated ACTH deficiency. We initiated corticosteroid therapy, and her gait disturbance improved promptly.

**Conclusion:**

Isolated ACTH deficiency may have major significance to the differential diagnosis of iNPH. Early consideration of this entity is anticipated to facilitate making an early diagnosis.

## Background

Isolated adrenocorticotropic hormone (ACTH) deficiency causes adrenal insufficiency as a result of impaired secretion of ACTH but no other anterior pituitary gland hormones. ACTH secretory cell damage, resulting from neurohypophysitis and an autoimmune mechanism, has been implicated in the etiology of this disorder [1]. Definitive diagnosis is relatively simple using serum ACTH measurement and pituitary stimulation tests [2], however, early diagnosis is not always easy because symptoms of adrenal insufficiency such as hypoglycemia and depressive state are nonspecific, and can be misdiagnosed as mental disorders [3]. The number of reports regarding isolated ATCH deficiency has increased along with the popularization of ACTH measurement, and in the field of cerebral/ neurological medical treatment, this disorder should be kept in mind when making a differential diagnosis. We experienced a case of isolated ATCH deficiency presenting with gradually progressive gait disturbance that needed to be distinguished from other forms of idiopathic normal pressure hydrocephalus (iNPH). We describe this case herein, with a review of the relevant literature.

## Case presentation

The patient was a 65-year-old woman who had undergone surgery at 19 years of age for appendicitis. Her medical history was otherwise unremarkable. Starting 3 years prior to the current presentation, the patient had gradually developed a gait disturbance, for which she consulted a local orthopedic surgery department, and upon diagnosis of knee osteoarthrosis received conservative therapy. At approximately 1 week before admissions, she had begun to experience loss of appetite and vomiting, and consulted a local physician. On suspicion of gastrointestinal disease, a thorough examination of the gastrointestinal tract was performed, but no organic disease was identified. Although the loss of appetite and vomiting resolved spontaneously, the gait disturbance gradually worsened, and magnetic resonance imaging (MRI) of the head revealed ventricular enlargement. The patient was thus referred to our department for suspected iNPH related to the gait disturbance.

**Fig. 1:**
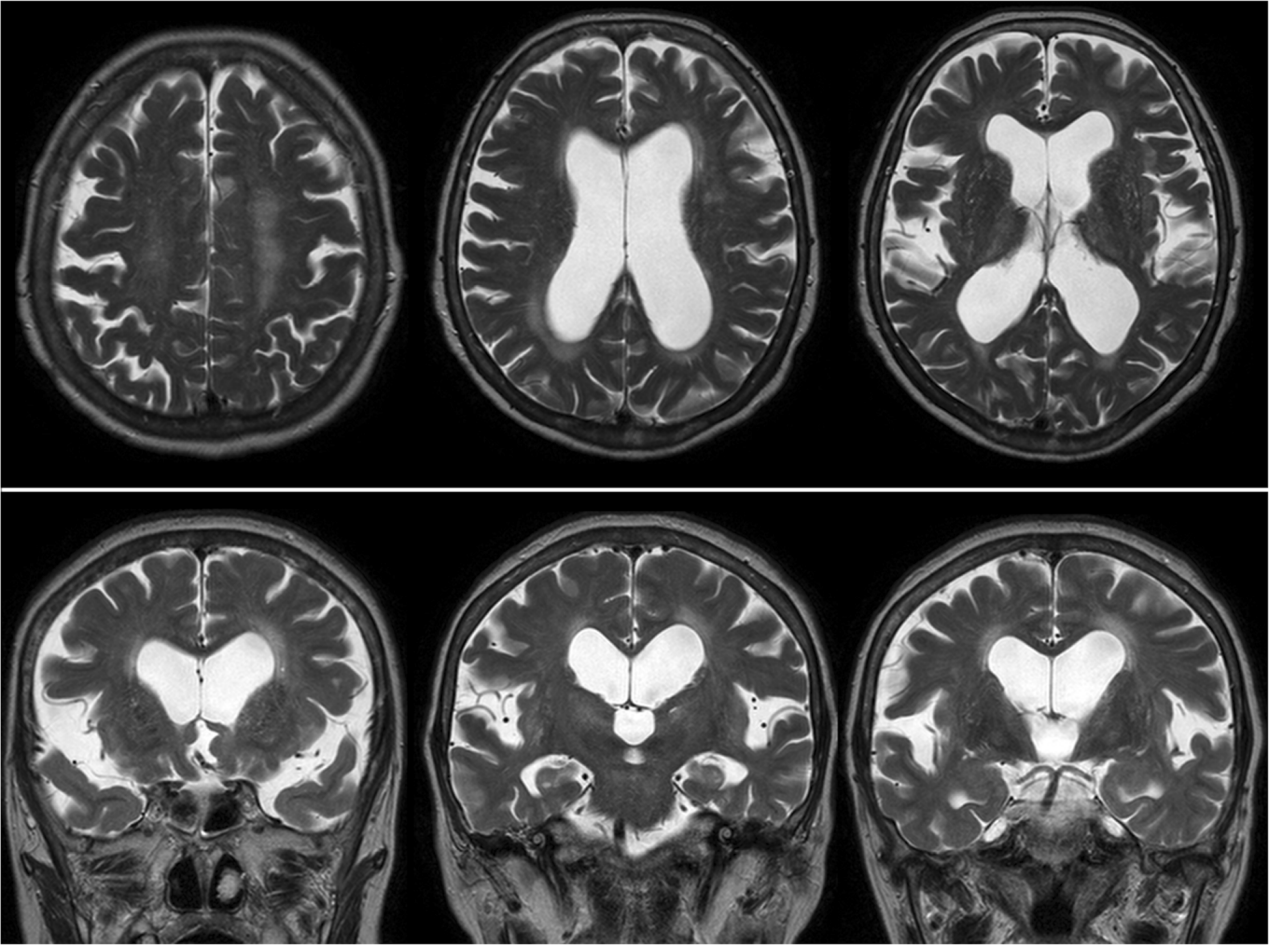
Upper: T2-weighted axial MRI revealed enlarged ventricles. Lower: In T2-weighted coronal MRI also showed enlarged ventricles, however, the major feature of disproportionately enlarged subarachnoid-space was not obvious

**Fig. 2:**
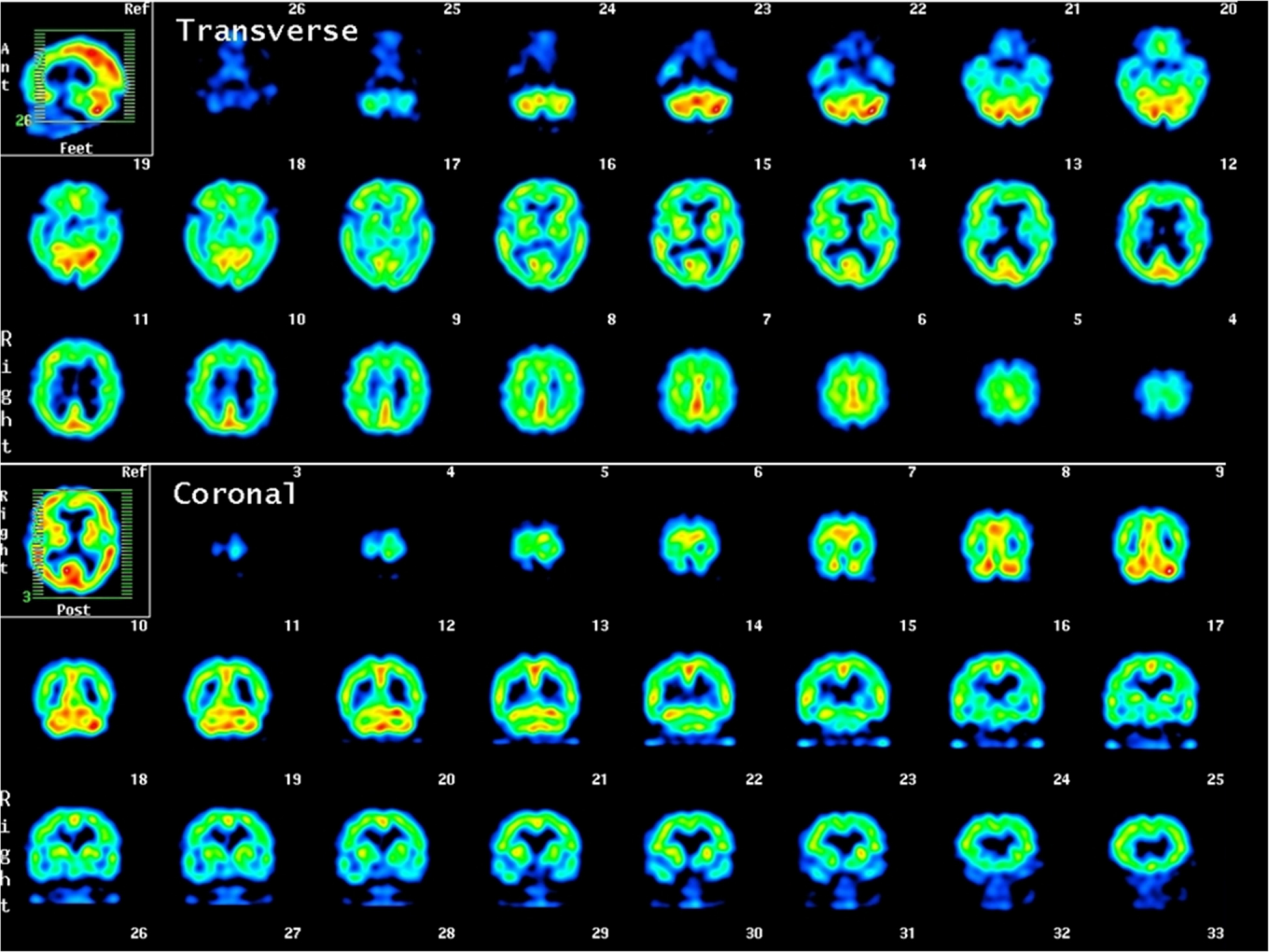
Single-photon emission computed tomography revealed areas of relatively decreased blood perfusion around the Sylvian fissure, while increased blood perfusion in the external layer around the convexity side

Her height of 148.3 cm, weight of 54.4 kg, and body mass index of 24.8 were all within standard ranges. There was no medical history that could have led to secondary hydrocephalus. There were no abnormalities in vital signs with body temperature 36 °C, blood pressure 110/63 mm/Hg, and heart rate 83 beats per minute. Although the patient’s consciousness was clear, 15 on the Glasgow Coma Scale (GCS), and there was no muscle weakness, she walked with her legs apart in short quick steps, and required a walking aid. While muscle tonus was normal, the patient complained of mild pain when extending the knee. As to higher order brain functions, mini mental state examination (MMSE) results were 24/30, and the frontal assessment battery (FAB) results were 9/18. Blood test findings at the time of admission were within normal range apart from mild hypoalbuminemia (total protein 4.6 g/dL, albumin 2.7 g/dL, total cholesterol 147 mg/dL, and LDL cholesterol 100 mg/dL), and elevated thyroid free T3 (TSH 4.333 μIU/mL, free T3 3.78 pg/mL, free T4 1.16 ng/dL). There were no blood-sugar or electrolyte (Na 140 mmol/L, Cl 106 mmol/, K 4.1 mmol/L, serum glucose 73 mg/dL) abnormalities. Head MRI revealed enlargement of all cerebral ventricles, with an Evan’s index of 0.4, and the Sylvian fissure appeared wide open consistent with iNPH. However, the specific feature of narrowing of the sulci at the high convexity area was not obvious (Fig. 1). Single-photon emission computed tomography revealed reduced cerebral blood flow (CBF) surrounding the Sylvian fissure, with relatively increased CBF in the convexity, which was consistent with the pattern for iNPH (Fig. 2). Findings for the sulci at the high convexity area were inconsistent with typical iNPH, however, and we thus reconsidered the possibility of other diseases that may have caused the gait disturbance.

**Fig. 3:**
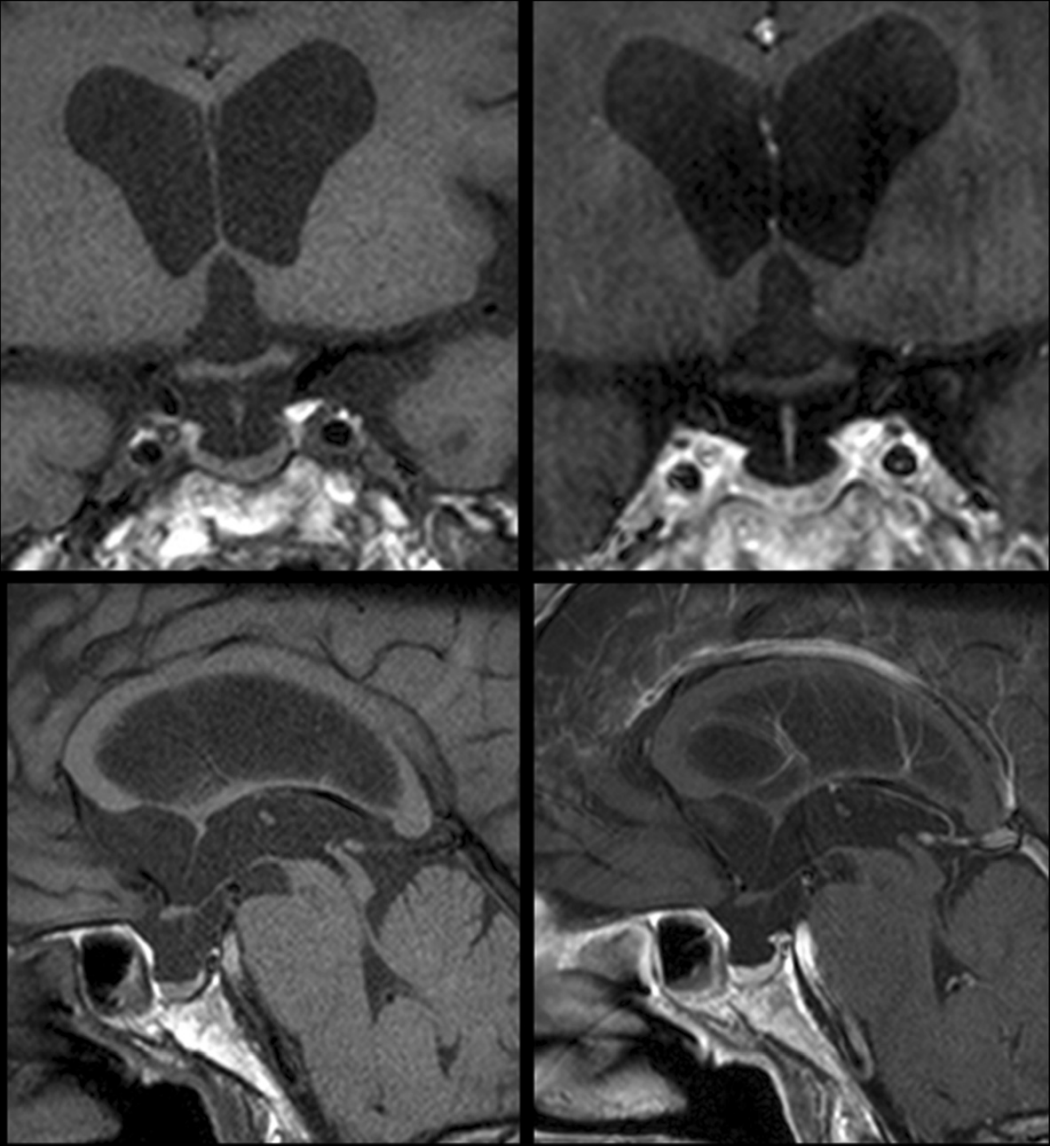
MRI showed normal pituitary gland measuring 12 mm in maximum diameter with no deviation of stalk, and there is no tumor or inflammatory lesion around the hypothalamic-pituitary area. (Upper left: T1 coronal MRI, Upper right: T1 gadolinium coronal MRI, Upper right: T1 gadolinium sagittal MRI, lower left: T1 gadolinium sagittal MRI)

**Fig. 4:**
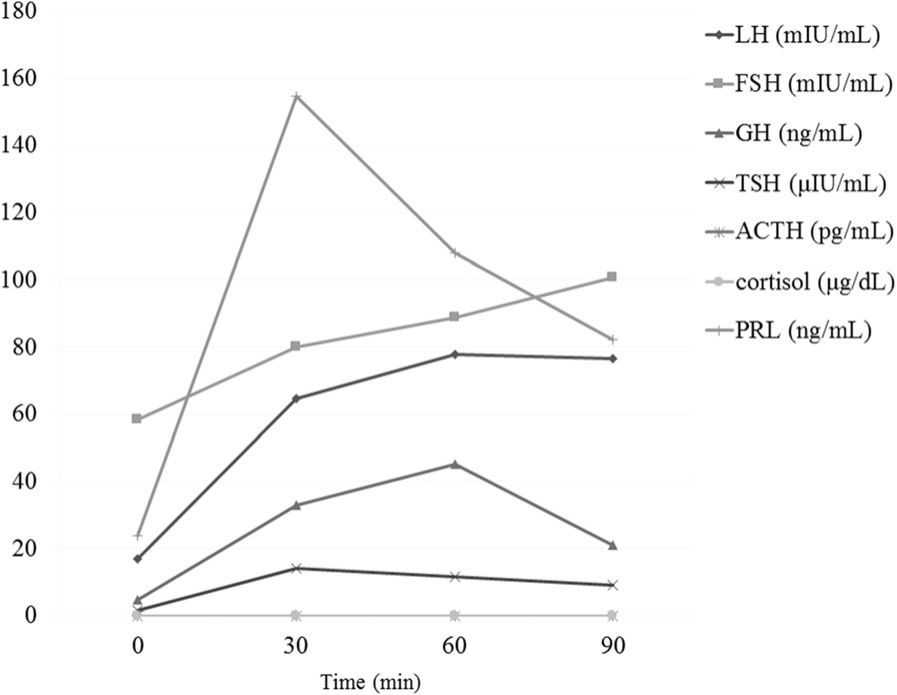
Pituitary stimulation test using corticotropin-releasing hormone, thyrotropin-releasing hormone, luteinizing hormone-releasing hormone and growth hormone releasing hormone showed no response in plasma cortisol level and ACTH level, while other hormones showed adequate response

Detailed tests were ultimately performed after hospital admission including a spinal tap. Following admission, the patient gradually became less active, and tended to stay in bed. Re-examination revealed blood sugar levels of 40 mg/dL and systolic blood pressure of 90 mm/Hg. On suspicion of impaired anterior pituitary function, scans were performed of the pituitary in the diencephalon, and baseline pituitary hormone levels were verified. The spinal tap results were negative. On MRI, pituitary gland was normal measuring 12 mm in maximum diameter with no enlargement or deviation of stalk, and there were no neoplastic lesions, inflammatory lesions in the pituitary area of the diencephalon, or changes suggesting that surgery had been performed in all sequences including contrast-enhanced dynamic images (Fig. 3). Table 1 presents baseline hormone levels, and Table 2 the results of the anterior pituitary gland hormone loading test using corticotrophin releasing hormone, thyrotropin releasing hormone, luteinizing hormone releasing hormone, and growth hormone releasing hormone. Pituitary hormone results included ACTH <1.0 pg/ml (normal range: 7.2–63.3), and cortisol levels <1.0 μ/dL (normal range: 4.0–18.3), indicating pituitary adrenal insufficiency. There were no decreases in other hormone levels, with relatively mild elevations of growth hormone, prolactin and free T3 levels. We checked carefully again the medical history of the patient, but there were no recent history of exogenous glucocorticoid treatment. An additional pituitary stimulation test was performed, in which ACTH and cortisol responses only were found to have disappeared, and isolated ACTH deficiency was thus diagnosed (Fig. 4). After initiation of hydrocortisone supplements, her gait quickly improved. While observing the clinical symptoms, hydrocortisone (Cortril ®) was adjusted to a daily dose of 15 mg, and after instructing the patient as to how to respond on sick days, she was discharged to return home. At approximately 6 months after discharge, although the ventricular enlargement on MRI was same as before, the patient scored 27/30 points on the MMSE and 15/18 points on the FAB.

**Table 1:** Endocrinogical findings about basal level of anterior pituitary hormone

TSH	4.333μIU/mL (0.350–4.940)
freeT3	3.78 pg/mL (1.71–3.71)
freeT4	1.16 ng/dL (0.70–1.48)
GH	1.85 ng/mL (0.28–1.64)
IGF-1	40 ng/mL (57–175)
LH	17.19 mIU/mL (5.72–64.31)
FSH	48.20 mIU/mL (<157.79)
PRL	34.61 ng/mL (4.91–29.32)
ACTH	<1.0 pg/mL (7.2–63.3)
Cortisol	≤1.0 μg/dL (4.0–18.3)

*TSH* thyroid stimulating hormone, *IGF* insulin-like growth factor, *LH* luteinizing hormone, *FSH* follicle stimulating hormone, *PRL* prolactin

(): Normal range of each test

**Table 2:** Endocrinogical findings about pituitary function test using CRH, TRH, LHRH and GRH

Time(min)	LH (mIU/mL)	FSH (mIU/mL)	GH (ng/mL)	TSH (μIU/mL)	ACTH (pg/mL)	Cortisol (μg/dL)	PRL (ng/mL)
0	17	58.35	4.77	1.563	<1.0	≤1.0	23.79
30	64.72	79.98	32.9	14.168	<1.0	≤1.0	154.7
60	77.72	88.88	45	11.643	<1.0	≤1.0	108
90	76.4	100.67	21.1	9.143	<1.0	≤1.0	82.13

*CRH* corticotropin-releasing hormone, *TRH* thyrotropin-releasing hormone, *LHRH* luteinizing hormone-releasing hormone, *GRH* growth hormone releasing hormone

## Discussion

Isolated ACTH deficiency is caused by damage to the ACTH-producing cells of the pituitary gland and can lead to secondary adrenocortical insufficiency. In 1954, Steinberg and colleagues were the first to report a patient presenting with general fatigue, weight loss, and hypoglycemia which improved after ACTH administration and their case was described as having true pituitary Addison’s disease [4]. This disorder has been called isolated ACTH deficiency. With the development and popularization of endocrine tests, the number of such reports has increasing in number.

The mean age at disease onset of patients withisolated ACTH deficiency is 50 years and the male to female is 1.2–3.6: 1, with the disease being slightly more common in men. In some instances, isolated ACTH deficiency is concurrent with primary hypothyroidism and Hashimoto’s disease. Furthermore, some cases may also be positive for anti-thyroglobulin antibodies and anti-pituitary antibodies [1], which implies the involvement of an autoimmune mechanism, though the details remain unclear. There have also been a few reports of cases associated with lymphocytic neurohypophysitis [5]. Other reported cases have presented with general fatigue, weight loss, and hyponatremia along with loss of olfaction following head trauma [6].

Isolated ACTH deficiency primarily presents with symptoms of adrenal insufficiency due to lack of ACTH secretion, along with general fatigue, loss of appetite, nausea, vomiting and skin dryness. However, when patients have psychological symptoms such as apathy and depression in addition to the above, the condition can be mistakenly diagnosed as a mental disorder. Thus, diagnosis on the basis of clinical symptoms is not necessarily easy [2]. Our case had symptoms resembling those experienced by patients with pituitary gland lesions of the diencephalon, which allowed us to make an early diagnosis.

In the presence of isolated ACTH deficiency, if low serum ACTH levels are confirmed, then a pituitary stimulation test will specifically exhibit the disappearance of ACTH only. Serum ACTH levels are usually below the sensitivity threshold of 5.0 pg/ml, though this may vary among reported cases. It is assumed that reported cases with ACTH deficiency also include patients with limited ACTH reserves, i.e. partially impaired ACTH secretion, which would be regarded as ACTH deficiency syndrome based on reports classifying disease severity [2].

Reported cases presenting with neurological symptoms of ACTH deficiency, excluding those in adrenal crisis with induced disturbance of consciousness and psychological symptoms, are relatively rare. There are reported cases presenting with elevated creatinine kinase and predominant proximal muscle weakness that required differential diagnosis from neuromuscular diseases, and that 2 years elapsed between the initial examination, prompted by serum ACTH levels measured incidentally, and definitive diagnosis [7]. The improvements of gait disturbance and cognitive dysfunction in this case are not interpreted in detail. It can be inferred that the replacement therapy cause any metabolic or circulating change as morphological change had not seem in this case. There are case reports describing isolated ACTH deficiency associated with dementia, in which CBF increased on positron emission tomography following hormone replacement therapy, while at the same time cognitive function improved [8].

Treatment for isolated ACTH deficiency involves replacement therapy, and as with more common forms of hypoadrenocorticism, a fixed amount of cortisol, a physiological glucocorticoid, is generally provided as a supplement. As with other adrenal insufficiency replacement therapies, it is vital to instruct patients that the medication dose should be increased at times of stress such as fever and infection.

The symptoms of isolated ACTH deficiency are non-specific, and diagnosis can be challenging. Cases have been reported in which diagnosis, including differential diagnosis from mental disorders, was difficult. In individuals age 60 years and older, iNPH presents with a triad of symptoms, i.e. gait disturbance, dementia, and urinary incontinence. Gait disturbance, which is a major cause of reduced activities of daily living, can be improved by shunt implantation and is a rare type of treatable dementia. However, gait disturbance and dementia are not limited to iNPH, instead being common in elderly individuals. Furthermore, iNPH is a disease in which caution is essential when differentiating the imaging findings occasionally seen in elderly individuals, such as ventricular enlargement due to atrophy of the cerebrum and cerebrovascular changes that develop with aging.

In the iNPH treatment guidelines, ventricular enlargement is defined by an Evan’s index of 0.3 or above, and although this is one criterion required to make the diagnosis of this disorder, an Evan’s index greater than 0.3 is seen in 3–4 % of healthy elderly individuals, and thus cannot be called a specific finding [9]. Characteristic findings of iNPH include a wide-open Sylvian fissure and narrowing of the sulci at the high convexity. In the present case, although iNPH was suspected on the basis of ventricular enlargement, no narrowing of the sulci was observed at the high convexity area. When the typical diagnostic findings of iNPH are not present, it is vital to keep isolated ACTH deficiency in mind when performing the differential diagnosis.

## Conclusion

Isolated ATCH deficiency can cause a variety of clinical symptoms. Hormone replacement therapy improves symptoms, and early diagnosis is closely related to patient prognosis. In patients suspected of having iNPH with an atypical clinical presentation and imaging findings, isolated ACTH deficiency should be considered early.

## Consent

Written informed consent was obtained from the patient for publication of this case report and any accompanying images. A copy of the written consent is available for review by the Editor of this journal.

### Ethical statement

This case report includes human date.

## Abbreviations

CBF: cerebral blood flow
FAB: frontal assessment battery
GCS: Glasgow coma scale
iNPH: idiopathic normal pressure hydrocephalus
MMSE: mini mental state examination
MRI: magnetic resonance imaging
